# Systematic investigation of TetR-family transcriptional regulators and their roles on lignocellulosic inhibitor acetate tolerance in *Zymomonas mobilis*

**DOI:** 10.3389/fbioe.2024.1385519

**Published:** 2024-03-22

**Authors:** Yubei Xiao, Tongjia Qin, Shuche He, Yunhao Chen, Han Li, Qiaoning He, Xia Wang, Shihui Yang

**Affiliations:** ^1^ State Key Laboratory of Biocatalysis and Enzyme Engineering, and School of Life Sciences, Hubei University, Wuhan, China; ^2^ Chinese Medicine College, Guangdong Yunfu Vocational College of Chinese Medicine, Guangzhou, Guangdong, China

**Keywords:** *Zymomonas mobilis*, TetR-family transcriptional regulator, RND-family efflux pump, acetate tolerance, RNA-seq, CRISPR-cas

## Abstract

TetR-family transcriptional regulators are widely distributed among bacteria and involved in various cellular processes such as multidrug and inhibitor resistance. *Zymomonas mobilis* is a industrial bacterium for lignocellulosic ethanol production. Although TetR-family regulators and their associated RND-family efflux pumps in *Z. mobilis* have been identified to be differentially expressed under various inhibitors and stressful conditions, there are no systematic investigation yet. In this study, bioinformatic analyses indicated that there are three TetR-family transcriptional regulators (*ZMO0281*, *ZMO0963*, *ZMO1547*) and two RND-family efflux pumps (*ZMO0282-0285*, *ZMO0964-0966*) adjacent to corresponding TetR-family regulators of ZMO0281 and ZMO0963 in *Z. mobilis*. Genetics studies were then carried out with various mutants of TetR-family regulators constructed, and *ZMO0281* was characterized to be related to acetate tolerance. Combining transcriptomics and dual-reporter gene system, this study demonstrated that three TetR-family regulators repressed their adjacent genes specifically. Moreover, TetR-family regulator ZMO0281 might also be involved in other cellular processes in the presence of acetate. In addition, the upregulation of RND-family efflux pumps due to *ZMO0281* deletion might lead to an energy imbalance and decreased cell growth in *Z. mobilis* under acetate stress. The systematic investigation of all three TetR-family regulators and their roles on a major lignocellulosic inhibitor acetate tolerance in *Z. mobilis* thus not only unravels the molecular mechanisms of TetR-family regulators and their potential cross-talks on regulating RND-family efflux pumps and other genes in *Z. mobilis*, but also provides guidance on understanding the roles of multiple regulators of same family in *Z. mobilis* and other microorganisms for efficient lignocellulosic biochemical production.

## Introduction

TetR-family transcriptional regulators (TFRs) are a large and important family of transcriptional regulators that are widespread among bacteria (Cuthbertson and Nodwell, 2013; Colclough et al., 2019). The name “TFR” is derived from the TetR protein, which is the first one discovered and characterized in details in *Escherichia coli* controlling the expression of *tetA* gene that encodes a tetracycline efflux pump responsible for antibiotic resistance (Anand et al., 2012; Moller et al., 2016). With more TFRs characterized, TFRs are implicated in the regulation of diverse physiological processes from primary metabolic processes to antibiotic production and resistance including virulence, osmotic stress, homeostasis, and biosynthesis of antibiotics and enzymes (Cuthbertson and Nodwell, 2013; Murarka et al., 2019; Muller et al., 2020; Su et al., 2022).

TFRs are originally known to act as transcriptional repressors (Ramos et al., 2005; Colclough et al., 2019), recent studies demonstrated that TFRs may also serve as activators or have dual functions (Pompeani et al., 2008; Murarka et al., 2019). Based on the location of TFRs relative to adjacent genes on the chromosome within 200 bp, TFRs can be classified into three types and these relationships can be used to roughly predict the target genes of the TFRs (Ahn et al., 2012): divergent orientation with neighbor (Type I), co-transcribed with an upstream or downstream neighbor with the intergenic DNA less than 35 bp separating them (Type II), neither I or II (Type III). In general, Type I TFRs are the most common ones (i.e., TetR regulating *tetA*). Both Type I and II TFRs are thought to act on local genes, whereas Type III TFRs act globally.

Although the number of TFRs continued to increase with the explosion of whole-genome sequencing projects, only a small fraction of this number has been characterized in details (Ahn et al., 2012; Long et al., 2020). TFRs usually function as a homodimer, which is constituted by two identical monomers that fold into 10 α-helices with connecting turns and loops. The common structure of each monomer recognizes two domains: an N-terminal DNA-binding domain with the typical helix-turn-helix motif (HTH) and a larger C-terminal regulatory core domain involved in dimerization and ligand binding. The N-terminal DNA-binding domains exhibit a high degree of sequence similarity and the most conserved region can be used as a regulator predictor of this family (Ramos et al., 2005; Agari et al., 2013; Deng et al., 2013). In contrast, the C-terminal regions are diverse possessing unique sequences, which allows different regulators in the family to accommodate specific sets of ligands. In most cases the C-terminal domains interact with one or more ligands, in turn altering the regulator’s ability to bind DNA (Wu et al., 2021). Strategies of phylogenomics, structural biology, and bioinformatics prediction based on metabolic pathways have been employed to identify ligands for TFRs of unknown functions (Le et al., 2011; Cuthbertson and Nodwell, 2013). Currently, a wide variety of TFRs ligands have been identified, including antibiotics, cell-cell signaling molecules, carbohydrates, proteins, metal ions, as well as fatty acids and their derivatives (Cuthbertson and Nodwell, 2013; Wu et al., 2021). The diversity of ligands suggests the diverse roles for TFRs in regulating cellular processes to be unraveled.

*Zymomonas mobilis* is an ethanologenic bacterium with many excellent physiological and industrial characteristics such as high-specific ethanol productivity and yield, high alcohol tolerance, and broad ranges of temperature and pH (Xia et al., 2019). Various genome-editing tools including endogenous and exogenous CRISPR-Cas editing systems have been developed for efficient genome engineering (Shen et al., 2019; Zheng et al., 2019), and a high-quality genome-scale *in silico* metabolic model was constructed to guide rational pathway design (Wu et al., 2023). Various recombinant *Z. mobilis* strains have also been constructed to produce platform biochemicals such as lignocellulosic ethanol, lactate, acetoin, isobutanol, 2,3-butanediol (2,3-BDO), and poly-3-hydroxybutyrate (PHB) (Yang et al., 2021; Li et al., 2022; Bao et al., 2023; Hu et al., 2023).

Previous studies demonstrated that TFRs *ZMO0281* and *ZMO1547* were upregulated and more abundant under ethanol stress (He et al., 2012a). In addition, ZMO0281 was also upregulated while *ZMO0285* gene encoding a resistance-nodulation-cell division (RND)-family efflux pump downregulated under furfural stress (He et al., 2012b). Further study in furfural resistance showed that either knockout of *ZMO00282*, *ZMO0283* or *ZMO0285* or overexpression of the repressor *ZMO00281* to downregulate the expression of *ZMO0282*, *ZMO0283* or *ZMO0285* conferred furfural tolerance (Yang et al., 2014b). However, *ZMO0283* and *ZMO0285* as well as *ZMO0964* were upregulated when either glucose or xylose was used as the carbon source for xylose-utilizing recombinant strain *Z. mobilis* 8b during short-term furfural shock (Yang et al., 2020). The upregulation of the *ZMO0282*-*ZMO0285* was observed as well in the xylose-utilizing recombinant strain *Z. mobilis* 2032 in synthetic hydrolysates containing various 25 lignocellulose-derived inhibitors (Zhang et al., 2019). Moreover, both *ZMO0282* and *ZMO0283* were upregulated in response to phenolic aldehydes (Yi et al., 2015). All these results suggested that the roles of TetR-family regulators and corresponding RND-family efflux pumps on stress tolerance of *Z. mobilis* are complicated and need further investigations. In this study, the relationships among TetR-family regulators and their corresponding adjacent RND-family efflux pumps in *Z. mobilis* were systematically investigated, and the underlying molecular mechanisms of TFRs on a major lignocellulosic inhibitor acetate tolerance were also investigated to facilitate future rational design and construction of microbial cell factories for efficient lignocellulosic biochemical production.

## Materials and methods

### Strains and growth conditions

Bacterial strains and plasmids used in this study are listed in Additional file 1: Supplementary Table S1. *Z. mobilis* ZM4 (ATCC 31821) was used as the parental strain in this study. Generally, ZM4 strain and its derivatives were cultured in rich medium RMG2 (20 g/L glucose, 10 g/L yeast extract, 2 g/L KH_2_PO_4_) or RMG5 (50 g/L glucose, 10 g/L yeast extract, 2 g/L KH_2_PO_4_) at 30°C, 100 rpm. The medium used for phenotypic testing and transcriptomic study under acetate stress was supplemented with 210 mM acetate (50% cell growth inhibited) in RMG5 (RMGA). *E. coli* DH5α and Trans110 were used for plasmid construction and demethylation in this study. All *E. coli* strains were cultured at 37°C, 250 rpm, in Luria Bertani medium (LB: 10 g/L tryptone, 5 g/L yeast extract, 10 g/L NaCl). Antibiotics were added into the media as required following the concentrations of: spectinomycin 100 μg/mL, chloramphenicol 50 μg/mL, and kanamycin 100 μg/mL. All solid media were prepared with 1.5% agar added into the liquid media.

### Genetic manipulation and recombinant strain construction

Shuttle plasmid pEZ15A was used for gene overexpression in *Z. mobilis* using the native promoter of target gene. pEZ-Dual was used to identify the relationships of candidate TetR-family regulators with their corresponding adjacent promotors in *Z. mobilis* (Yang et al., 2019). To construct candidate promoters of P_*0282-0285*_ and P_*0964-0966*_ into the dual reporter-gene system, the P*tet* was replaced by two promoters individually. For plasmid construction, primers containing 15–25 nucleotides that overlap with adjacent DNA fragments were synthesized by TsingKe (Beijing, China) and used for polymerase chain reaction (PCR) to obtain target fragments. After purification with gel purification kit (TsingKe, Beijing, China), gene and vector fragments were ligated using the T5 exonuclease (NEB, WA, United States) by the Gibson Assembly method. After verification by colony PCR and Sanger sequencing, the correct recombinant plasmid was extracted and then transformed into *Z. mobilis* via electroporation (0.1 cm cuvette, 1.6 kV, 200 Ω, 25 μF) using a Gene Pulser^®^ (Bio-Rad, CA, United States). Recombinant cells were grown on RMG2 agar plate containing corresponding antibiotics. The correct transformants were screened by colony PCR.

Plasmid pL2R was used for gene deletion in *Z. mobilis* using the native CRISPR-Cas genome editing system (Zheng et al., 2019). For gene deletion, the interference plasmid was initially constructed with spacer. The spacer was designed 32-bp sequences immediately after a 5-NCC-3’ PAM in the target gene. Plasmid pL2R was digested with *Bsa* I at 37°C overnight. Two single-stranded oligonucleotides were annealed by firstly heating to 95°C for 5 min and then cooling down gradually to room temperature. The annealed spacer and the digested linear DNA vector were ligated by T4 ligase at 18°C overnight. Subsequently, Gibson Assembly method was utilized for donor construction. Donor DNA fragments containing extra ∼800 bp upstream and downstream flank sequences of target gene were amplified and then cloned into the corresponding interference plasmid by T5 exonuclease (NEB, WA, United States). The resultant editing plasmid was electroporated into *Z. mobilis* to delete the candidate gene. Finally, correct transformants with correct PCR results were cultivated in RMG2 medium without antibiotics at 30 °C and passaged for several generations until the pL2R editing plasmids were cured.

### Flask fermentation and cell growth measurement

*Z. mobilis* and derived strains from frozen glycerol stocks were revived in 10 mL glass bottles containing 5 mL RMG5. After cultivated overnight without shaking at 30°C, the culture was transferred into 50 mL flask containing 40 mL RMG5 medium and cultured for 8–10 h without shaking to the mid-log phase as the seed culture. Then the seed culture was harvested and inoculated in fresh 40 mL RMG5 or RMGA medium with an initial OD_600_ nm value of 0.1. During the fermentation, the cultures were collected at different time points and cell growth was determined by measuring the optical density at 600 nm with a UV-visible spectrophotometer (UV-1800, AOE, China).

### Bioinformatic analyses

Blast was used to predict TetR-family transcriptional regulators and their corresponding RND-family efflux pumps within the genome of *Z. mobilis*. The 3-dimensional structures of the candidate TetR-family transcriptional regulators, ZMO0281, ZMO0963 or ZMO1547, were predicted by AlphaFold2 online server ColabFold (https://colabfold.mmseqs.com) (Mirdita et al., 2022). The secondary DNA structures of the ZMO0281-ZMO0282, ZMO0963-ZMO0964 and ZMO1547-ZMO1545 intergenic region, which were recognized as the dual promoter correspondingly, were analyzed by GeneQuest software, and the palindromic sequence was predicted using Web 3DNA 2.0 (http://web.x3dna.org/index.php/rebuild) (Li et al., 2019). The probable TetR-family regulator binding site was then recognized by the ScanProsite tool (https://prosite.expasy.org/scanprosite/) with the known binding sites of TetR-family regulators in EBI database (https://www.ebi.ac.uk/interpro/entry/InterPro/IPR036271/). The molecular docking studies of the TetR-family regulators to different predicted DNA palindrome sequences were performed using HADDOCK2.4 software (https://wenmr.science.uu.nl/haddock2.4/) (van Zundert et al., 2016; Honorato et al., 2021), repaired by the RepairPDB module of foldx 5.0 (Delgado et al., 2019), and finally the interaction energy was calculated by AnalyseComplex. All structures were visualized by Discovery Studio 2021 (Dassault Systèmes, Shanghai, China).

### RNA-seq transcriptomic study

Cells cultured to mid-log phase were collected by centrifuging at 1522 x g. According to standard Illumina protocols, RNA-Seq was carried out by the Beijing Genomics Institute (BGI, Wuhan, China). RNA-Seq FastQ data passed the quality control evaluated by FastQC program were imported into CLC Genomics Workbench (version 14.0, Invitrogen, CA, United States) for reads trimming and RNA-Seq analysis to get the RPKM value (reads mapping to the genome per kilobase of transcript per million reads sequenced) of each gene. Significantly differentially expressed genes were determined with a cut-off of log_2_-fold change value of 1, and only those with *p*-value ≤0.05 were considered for further analysis. The raw sequence data were submitted into NCBI Sequence Read Archive (SRA) database (BioProject accession numbers: PRJNA1047928).

### Characterization of TetR-family regulation *in vivo* by flow cytometry analysis

The regulation of TetR-family regulators to candidate adjacent promotors was detected in terms of fluorescence intensity using the dual reporter-gene system established previously in *Z. mobilis* (Yang et al., 2019). Recombinant strains cultured to mid-log phase were collected, washed with 1X phosphate-buffered saline (PBS: 8.0 g/L NaCl, 0.2 g/L KCl, 1.44 g/L Na_2_HPO_4_, 0.24 g/L KH_2_PO_4_, pH 7.4) twice and resuspended into 1X PBS to a final concentration of 10^7^ cells/mL. Cells were analyzed by flow cytometry using Beckman CytoFLEX FCM (Beckman Coulter, CA, United States) with 1X PBS as the sheath fluid. The cell fluorescence of EGFP was excited with the 488 nm and detected with FITC, and opmCherry was excited with the 561 nm and detected with PC5.5. Compensation was applied to ensure that the EGFP has minimal impact on the detection of mCherry with at least 20,000 events of each sample analyzed. Data were processed via FlowJo 10 software (FlowJo, LLC, United States). The mean fluorescence intensity of triplicates was calculated, then the ratio of average EGFP/average opmCherry was used to analyze the interaction of TetR-family regulator and candidate promotor. Data presented in the graphs are the mean ± SD of triplicates calculated by the GraphPad Prism statistical software (version 8.3.0, GraphPad, CA, United States). *t*-test analysis was performed as needed and only *p*-value ≤0.05 was considered as statistically significant difference.

## Results and discussion

### Bioinformatic analyses of TetR-family regulators in *Z. mobilis*

**FIGURE 1:**
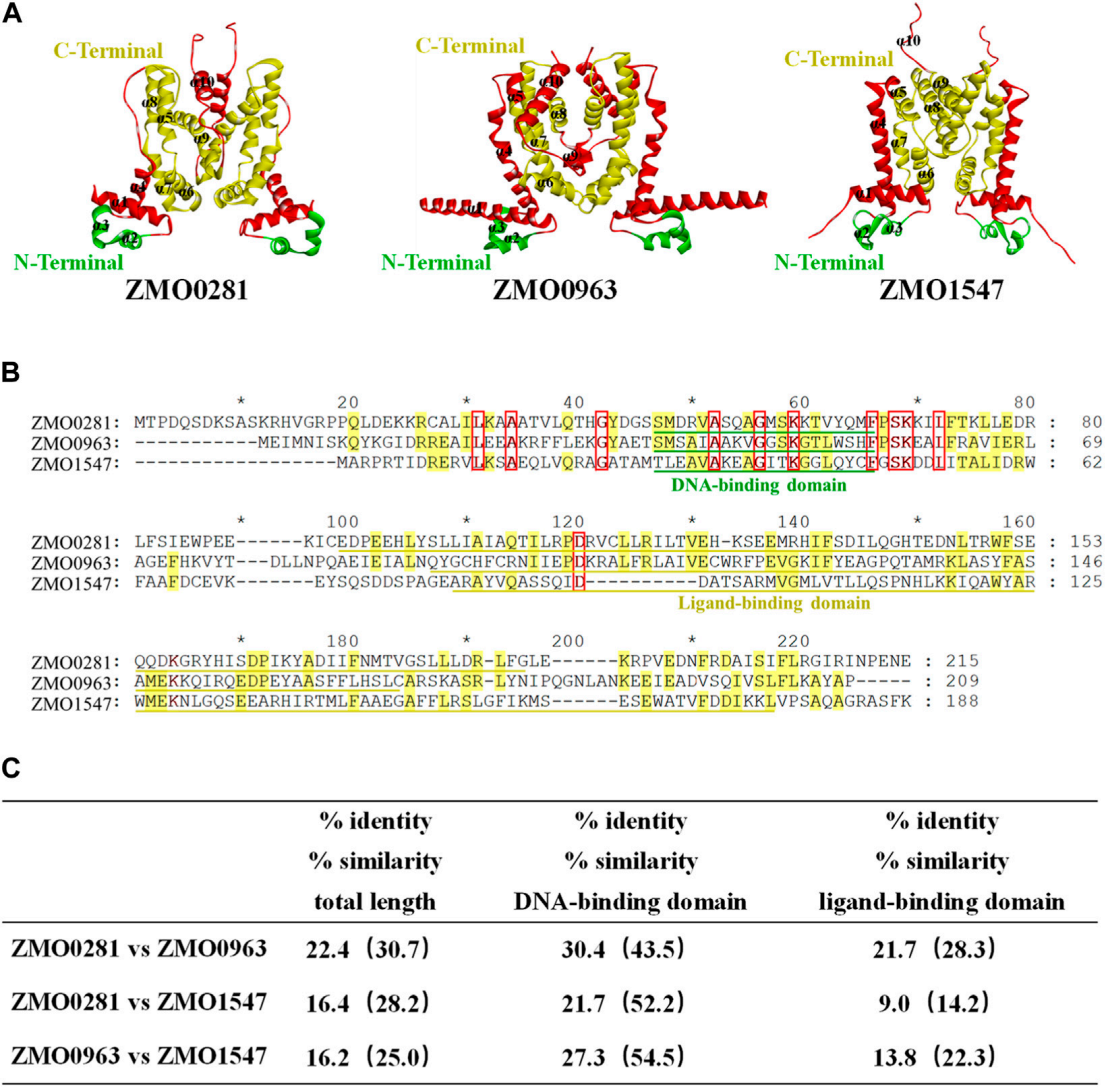
Structure prediction **(A)**, sequence alignment **(B)**, and similarity **(C)** of three TetR-family regulators ZMO0281, ZMO0963, and ZMO1547 in *Z. mobilis*. 3D structure of three TetR-family regulators predicted as a dimer, and the green residues in the structure indicates the DNA binding region of the protein and the yellow portion indicates the probable ligand binding region with N and C terminals marked, The parts outside these two functional areas were shown in red **(A)**. Sequence alignment of three TetR-family regulators. Conserved residues in all three TetR-family regulators are displayed by red frame with red bold font highlighted, while conserved residues in only two TetR-family regulators are in yellow-color highlighted; the DNA-binding domain of each protein is underlined in green color, and the ligand-binding domain is in yellow color **(B)**. Summary of amino acid identity and similarity among three TetR-family regulators **(C)**.

The genome sequence and annotation of *Z. mobilis* were released and further improved (Seo et al., 2005; Yang et al., 2009; Yang et al., 2018). Three genes *ZMO0281*, *ZMO0963*, and *ZMO1547* were annotated as the TetR-family transcriptional regulator in *Z. mobilis* ZM4, which are also analyzed using bioinformatic tools in this study. The predicted molecular weight of ZMO0281, ZMO0963, and ZMO1547 are 24.9 kDa, 23.8 kDa, and 20.9 kDa, respectively. The protein structure of each protein was predicted using AlphaFold2-ColabFold software and each of them contains 10 α-helices (Figure 1A). The structures were predicted as a dimer as all known TetR-family regulators, but were executed without templates, due to the poor protein sequence similarity (less than 30%) with TetR-family regulators with experimental crystal structures. The BLAST search also revealed that three proteins have a conserved helix-turn-helix motif (HTH) domain of the TetR-family transcriptional regulators with a consensus sequence (IPR001647) near the N-terminal region (marked in green), which comprises residues Ser46-Met65 in ZMO0281, Thr35-His54 in ZMO0963, and Met28-Cys47 in ZMO1547, respectively (Figure 1B).

However, the C-terminal region analysis showed that ZMO0281 and ZMO0963 contain the conserved ligand binding domain IPR039536, while ZMO1547 possesses a different domain IPR041479 (marked in yellow). Sequence alignment between these three TetR-family regulators also exhibited a moderate sequence similarity of protein ZMO0281 and ZMO0963 with 22.4% identity (30.7% similarity). This similarity is not limited to the DNA-binding domain (43.5% similarity) and extends through the ligand-binding domain (28.3% similarity). In contrast, ZMO0281 and ZMO0963 only share 16.4% and 16.2% identity with ZMO1547, respectively (Figure 1C). The bioinformatic analysis of three TetR-family regulators suggested that ZMO0281 has relatively high similarity to ZMO0963 and they may possess similar biological functions in *Z. mobilis,* where ZMO1547 could be different from them.

**FIGURE 2:**
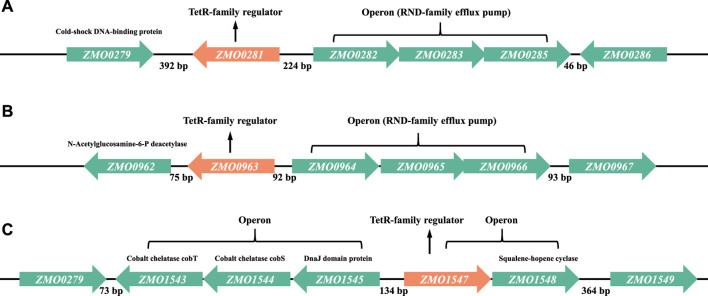
Detailed genetic maps of three TetR-family regulators of ZMO0281 **(A)**, ZMO0963 **(B)**, and ZMO1547 **(C)** in *Z. mobilis*. In each subfigure, the architecture and orientation of each gene are depicted. Each TetR-family regulator encoding gene is marked in orange color, other genes are marked in green color. Numbers represent the intergenic bases between the genes.

In most cases, TetR-family regulators were described to repress the genes often located in the same operon or the adjacent operon on the chromosome (Deng et al., 2013; Colclough et al., 2019). Therefore, the genomic location of each TetR-family regulator was investigated to identify the candidate target genes regulated by the TetR-family regulators ZMO0281, ZMO0963, or ZMO1547 (Figure 2). In *Z. mobilis* genome, a three-gene operon *ZMO0282-0285* encoding a RND-family membrane efflux system is located 224 bp upstream of *ZMO0281* and transcribed divergently. RND system is one of the major families of bacterial drug efflux pumps. *ZMO0279* is located in the opposite orientation 392 bp downstream of *ZMO0281* and annotated as a cold-shock DNA-binding protein (Figure 2A).

According to the criteria proposed by Ahn et al. (Ahn et al., 2012) that the majority of TetR-family regulators are separated from their divergent partners by 200 bp or less (the “200 bp” rule), ZMO0281 was supposed to control the divergently oriented adjacent operon *ZMO0282-0285*, consistent with the results reported before (Yang et al., 2014b; Zhang et al., 2019). Similar to ZMO0281, ZMO0963 was also predicted to regulate the expression of another RND-family efflux pump encoded by an operon of *ZMO0964-0966*, which is located 92 bp upstream of *ZMO0963* and divergently oriented (Figure 2B). Although *ZMO0962* gene has same orientation as *ZMO0963* (downstream), it is not likely to be co-transcribed with *ZMO0963* since the intergenic region separating them is 75 bp (>35 bp) and does not observe the rule of second group of TetR-family regulators (Ahn et al., 2012).

As shown in Figure 2C, three genes *ZMO1543*, *ZMO1544*, and ZMO*1545* are divergently oriented to TetR-family regulator *ZMO1547* with a 134 bp intergenic upstream DNA sequence. These three divergent genes are thought to form an operon supporting the stress resistance functions with *ZMO1543* and *ZMO1544* encoding cobalt chelatase subunit CobT and CobS, and ZMO*1545* encoding heat shock protein DnaJ domain protein. In addition, *ZMO1547* is likely to be co-transcribed with a downstream neighbor gene *ZMO1548*, which encodes squalene-hopene cyclase. Combining with the genomic information, ZMO1547 was speculated to control the transcription of the *ZMO1547*- *ZMO1548* operon or the *ZMO1543*-ZMO*1545* operon or both in *Z. mobilis*.

TetR-family regulators usually bind their operator sequences composing of 10–30 bp palindromic sequences to control the target genes. Based on the genomic configuration of TetR-family regulators and their corresponding potential target genes in *Z. mobilis*, the intergenic regions of *ZMO0281*-*ZMO0282*, *ZMO0963*-*ZMO0964* and *ZMO1545*-*ZMO1547* were analyzed by Web 3DNA 2.0 software to identify the DNA binding sites. As expected, three pairs of palindromic repeats were identified as potential operators correspondingly, which were named as “ZMO0281_O1” and “ZMO0281_O2” in *ZMO0281*-*0282* for ZMO0281, “ZMO0963_O1” and “ZMO0963_O2” in *ZMO0963*-*ZMO0964* for ZMO0963, and “ZMO1547_O1” and “ZMO1547_O2” in *ZMO1545*-*ZMO1547* for ZMO1547 (Supplementary Figure S1). The six potential palindromic repeats were also performed in molecular docking analysis by HADDOCK2.4 software to confirm the interactions with the TetR-family regulators. As shown in Supplementary Figure S1, only three of the molecular docking energy values were positive (ZMO0281 binding to “ZMO1547_O1” and “ZMO1547_O2,” and ZMO0963 binding to “ZMO1547_O2”), the energy value of other 9 combinations were negative. The results showed that all three regulatory genes could theoretically bind to promoter regions of two RND families although experimental work is needed to examine and confirm the regulation of TetR-family regulators in *Z. mobilis*.

### Characterization of TetR-family regulators in *Z. mobilis*

The genomic map of TetR-family regulators suggested that they may control the expression of their corresponding RND-family efflux pump genes, which emphasized a potential role of these regulators in stress responses. Previous studies in *Z. mobilis* also demonstrated that genes encoding TetR-family regulators and their corresponding RND-family efflux pumps were differentially regulated by the toxic stressors (He et al., 2012a; He et al., 2012b; Yang et al., 2014b; Yi et al., 2015; Zhang et al., 2019; Yang et al., 2020). In this study, the biological functions of all three TetR-family regulators response to acetate stress were systematically examined. The deletion mutants of each TetR-family regulator were constructed as well as the corresponding complementary and overexpression mutants, and the cell growth of these mutant strains were investigated in normal RMG5 medium as well as under 210 mM acetate stress.

**FIGURE 3:**
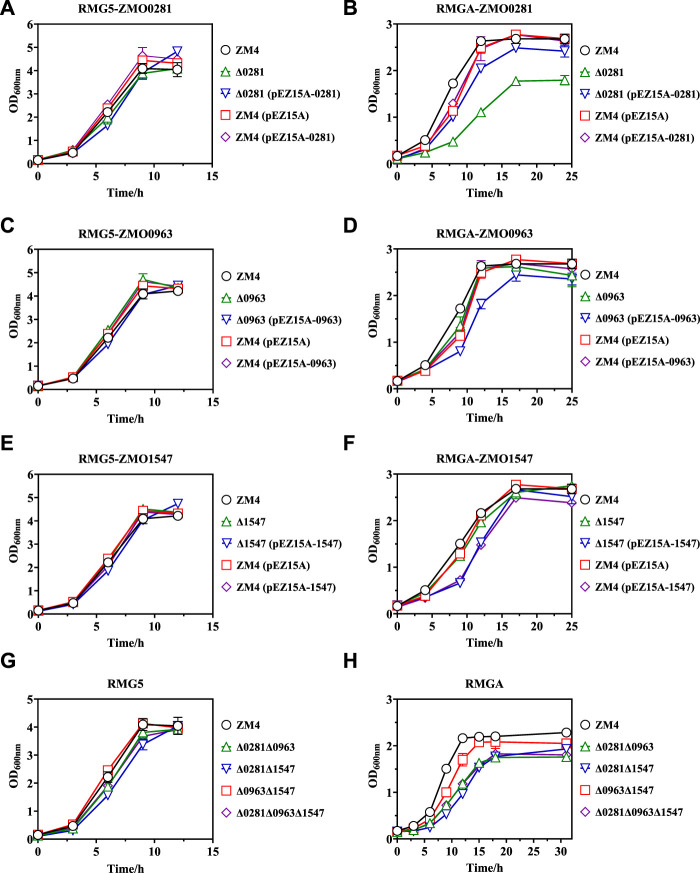
Cell growth of different *Z. mobilis* strains under normal RMG5 medium **(A,C,E,G)** and 210 mM acetate stress RMGA medium **(B,D,F,H)**. *ZMO0281* deletion mutant strains Δ0281, complementary strain Δ0281 (pEZ15A-0281), and over-expression strain ZM4 (pEZ15A-0281) compared with the control strains of wild-type ZM4 or ZM4 (pEZ15A) containing the empty plasmid pEZ15A, respectively **(A,B)**. *ZMO0963* mutant strains compared with ZM4 and ZM4 (pEZ15A) **(C,D)**. *ZMO1547* mutant strains compared with ZM4 and ZM4 (pEZ15A) **(E,F)**. Deletion mutant strains with two or three TetR-family regulator genes deleted compared with ZM4 **(G,H)**. Cell growth was monitored by measuring OD_600_ at indicated time points.

Cell growth results demonstrated that the control strains of ZM4 and ZM4 (pEZ15A) as well as recombinant strains of Δ0281, Δ0281 (pEZ15A-0281), and ZM4 (pEZ15A-0281) showed almost identical growth rates in RMG5 medium (Figure 3A). Similar cell growth was also observed in *ZMO0963* and *ZMO1547* related mutant strains (Figures 3C, E), which is consistent with previous reports (Cuthbertson and Nodwell, 2013; Boutrin et al., 2016; Su et al., 2022). When exposed to acetate, all strains were severely inhibited under acetate compared with those without acetate supplementation (Figures 3B, D, F), which is also agreed with previous studies in *Z. mobilis* (Yang et al., 2010; Yang et al., 2014a; Yang et al., 2014c).

Among three TetR-family regulators, the disruption of *ZMO0281* further dramatically decreased cell growth with the maximum biomass of mutant strain Δ0281 dropped from 2.63 to 1.79 compared to ZM4 at 17 h post-inoculation, which was 5 h longer than that of ZM4. The reduced cell growth can be restored by gene complementation, and the final OD_600_ value of the complementary strain Δ0281 (pEZ15A-0281) can be achieved to 2.42. However, compared with the control strain ZM4 (pEZ15A) containing the empty vector pEZ15A, the overexpression strain of ZM4 (pEZ15A-0281) did not exhibit any growth advantage on acetate tolerance (Figure 3B). Different from *ZMO0281*, the *ZMO0963* deletion mutant strain Δ0963 showed slightly decreased cell growth compared with ZM4, while its gene complementary strain Δ0963 (pEZ15A-0963) extended the stationary phase to 17 h with the maximum OD_600_ value of 2.44. Almost no growth difference was detected between *ZMO0963* overexpression strain ZM4 (pEZ15A-0963) and the control strain ZM4 (pEZ15A) (Figure 3D). The cell growth performance of *ZMO1547* deletion mutant Δ1547 and complementary strain Δ1547 (pEZ15A-1547) was similar to *ZMO0963* mutants, but its overexpression strain ZM4 (pEZ15A-1547) affected cell growth negatively (Figure 3F).

Cell growth results of all the TetR-family regulator mutants suggested that *ZMO0281* in *Z. mobilis* may play an important role on acetate tolerance, and other two TetR-family regulators *ZMO0963* and *ZMO1547* had little influence on acetate tolerance. To further confirm the roles of TetR genes in acetate response, four mutant strains of Δ0281Δ0963, Δ0281Δ1547, Δ0963Δ1547, and Δ0281Δ0963Δ1547 were constructed to delete two or all three TetR-family regulator genes, respectively. Similar to single gene deletion, only slight growth decrease was observed for these four mutants in normal condition compared with the control ZM4 (Figure 3G), but displayed a reduced cell growth than ZM4 under acetate stress (Figure 3H). Among these four strains, three mutants Δ0281Δ0963, Δ0281Δ1547, and Δ0281Δ0963Δ1547 with *ZMO0281* deletion mutant showed similar cell growth to that of the single *ZMO0281* deletion mutant Δ0281, and were obviously slower than that of ZM4 with a final OD_600_ value around 1.80 achieved 18 h post-inoculation (Figures 3B, H). Cell growth of Δ0963Δ1547 mutant containing *ZMO0281* was reduced slightly, which further suggested that ZMO0281 involved in acetate tolerance in *Z. mobilis*.

Previous study in *Z. mobilis* confirmed that overexpression of ZMO00281 conferred the furfural tolerance (Yang et al., 2014b), which is different from the results in present work. It suggested TetR-family regulator ZMO00281 may have various roles on responding to different stressors. Besides acetate, other weak monocarboxylic acids, such as formic and levulinic acids, are presented as well in the lignocellulosic hydrolysate, which also inhibit microbial cell growth. Moreover, some organic acids, such as lactic, succinic, 3-hydroxy propionic, and itaconic acids are produced by engineered *Z. mobilis* strains. Current work on ZMO00281 responding to acetate may provide guidance on reduce the stress of these organic acids in *Z. mobilis*.

### Examination of TetR-family regulators on the transcription of their adjacent genes

To evaluate the effect of TetR-family regulators on the transcription of their adjacent genes as well as their roles on acetate response, RNA-Seq transcriptomics was performed in four mutants of Δ0281Δ0963, Δ0281Δ1547, Δ0963Δ1547, and Δ0281Δ0963Δ1547 cultured in RMG5 with or without 210 mM acetate supplementation. The differentially expressed genes (DEGs) were identified through analysis of variance (ANOVA) using strains and media as the variables, and a total of 533 DEGs were detected for subsequent detailed analyses (Supplementary Table S2).

**FIGURE 4:**
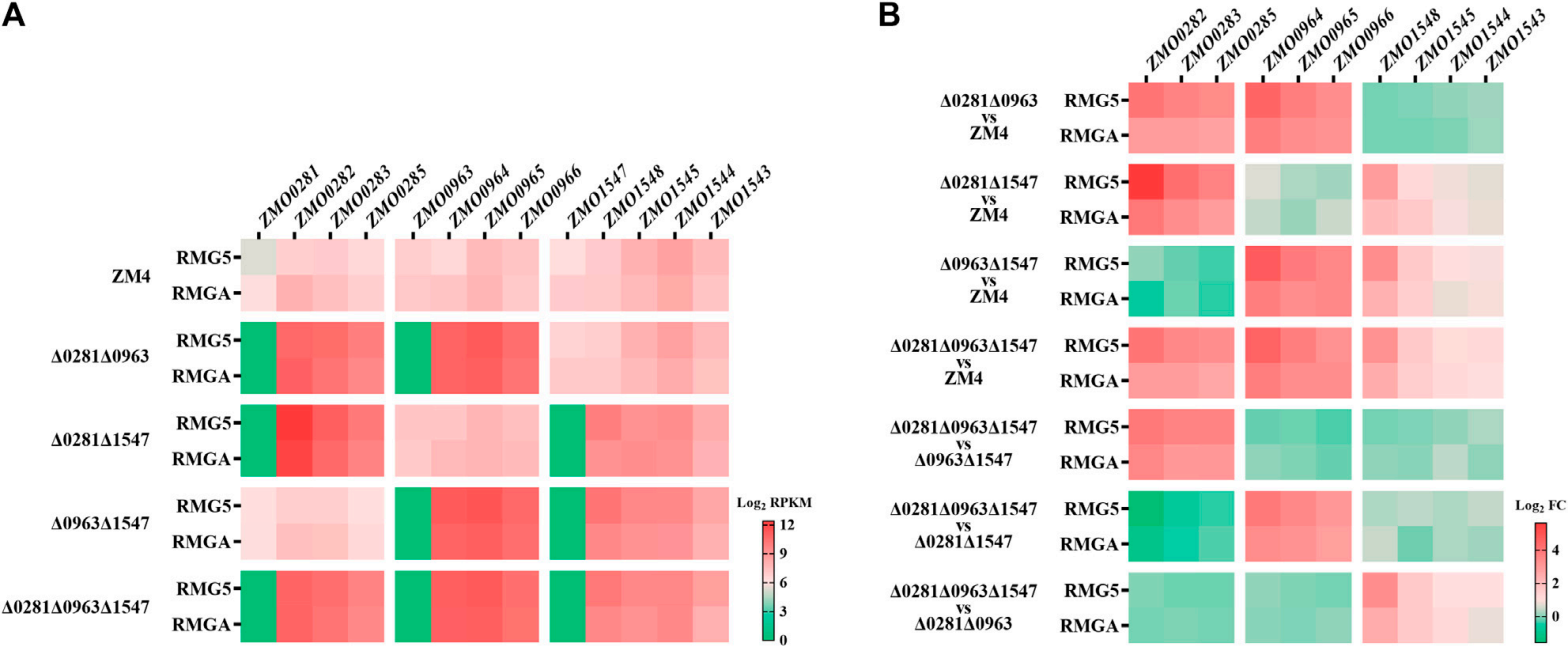
Transcriptional analysis examining the regulation of TetR-family regulators to their adjacent genes by RNA-Seq. Transcriptional levels of each gene in the wild-type ZM4 and four TetR mutant strains cultured in RMG5 or RMGA **(A)**, as well as fold changes of the transcriptional levels **(B)**. All RNA-Seq data were log_2_RPKM values. Red square represents positive RPKM values, green square represents little or no mRNA detection in A, while red and green squares represent the increased and repressed gene expression respectively in **(B)**.

The transcriptional levels of TetR-family regulator genes were consistent with the strain background with no mRNA of corresponding TetR-family regulators detected (Figure 4A), which confirmed that the TetR-family regulator genes were completely deleted. The transcriptional levels of the RND-family efflux pump operon *ZMO0282-0285* that is divergently oriented adjacent to *ZMO0281* were remarkably enhanced in the *ZMO0281* deletion mutants cultured in RMG5 (Figures 4A, B; Supplementary Tables S2-2), including mutant strains of Δ0281Δ0963, Δ0281Δ1547, and Δ0281Δ0963Δ1547 compared with ZM4, as well as Δ0281Δ0963Δ1547 compared with Δ0963Δ1547 (red square, >10-fold). The expression of *ZMO0282-0285* was enhanced under both normal and acetate stress conditions although the expression level under acetate stress had a moderate enhancement (>6-fold). These results clearly indicated that the adjacent RND-family efflux pump operon *ZMO0282-0285* was negatively controlled by TetR-family regulator ZMO0281.

The negative regulation of ZMO0281 to its adjacent genes was detected as well in another TetR-family regulator ZMO0963, with the transcriptional levels of its divergent neighbor operon *ZMO0964-0966* induced dramatically in strains lacking *ZMO0963* in both normal and acetate stress conditions (Figures 4A, B; Supplementary Tables S2-2). Slightly different from ZMO0281 and ZMO0963, the mRNA levels of the flanked genes of *ZMO1547,* upstream adjacent operon *ZMO1543-ZMO1545* and downstream neighbor *ZMO1548*, were all increased in the *ZMO1547* deletion mutant strains with a moderate transcription enhancement of *ZMO1548*, which was >7-fold increase in RMG and > 4-fold increase in acetate stress conditions (Figure 4B), while ca 2-fold increase of the expression of *ZMO1543-ZMO1545* in two culture conditions. The induction of two flanking genes by *ZMO1547* indicated that TetR-family regulator ZMO1547 might negatively regulate the expression of *ZMO1548* and *ZMO1543-ZMO1545.* Together, these results demonstrated that all three TetR-family regulators in *Z. mobilis* negatively controlled corresponding adjacent genes, and such regulation were independent to other TetR-family regulator without detectable cross-talks.

A similar induced expression of the adjacent operons was observed in the deletion mutant of *ZMO0281* or *ZMO0963* (Figures 4A, B), while only a slight upregulation of the flanking genes was detected in *ZMO1547* deletion mutant background. These results were coherent to a high sequence similarity and similar genetic loci of *ZMO0281* and *ZMO0963* but was different from *ZMO1547* (Figures 1, 2), which suggested that there might be a unique regulation mechanism in ZMO1547 from those of ZMO0281 and ZMO0963.

Moreover, some other genes were also detected with enhanced expression levels similar to the genes adjacent to TetR-family regulators, including *ZMO0399* whose expression levels fluctuated similar to the RND-family operon *ZMO0282-0285* as well as *ZMO0798-0801* operon, *ZMO0967-0968,* and gene *ZMO1612* whose expression levels fluctuated similar to the RND-family operon *ZMO0964-0966*. While, there were no genes detected with similar fluctuated expression levels to *ZMO1543-1545* and *ZMO1548* (Supplementary Tables S2-1). The promoter regions of these genes were then analyzed by Web 3DNA 2.0 software as well to identify the palindromic sequences and compared with those in the promoter of corresponding RND-family operon genes. The results revealed that few common sequences were shared between them individually. Accordingly, the TetR-family regulators in *Z. mobilis* most likely control their corresponding adjacent genes, which belong to the majority of TetR-family regulators according to the criteria proposed by Ahn et al. (2012).

A flow cytometry-based dual reporter-gene system, which has been proved to be an effective tool to characterize genetic elements including the promoters in *Z. mobilis* (Yang et al., 2019), was applied in this study to examine the regulatory relationship of the TetR-family regulators with their potential regulatory adjacent genes. The intergenic region *ZMO0281*-*ZMO0282*, *ZMO0963*-*ZMO0964* were amplified as the promoter P_*0282-0285*_ and P_*0964-0966*_ respectively and then inserted in the system to control the reporter gene *EGFP* for quantification with reporter gene *opmCherry* controlled by constitutive promoter P*lacUV5* for calibration (Supplementary Figure S2). The ratio of EGFP fluorescence intensity versus the opmCherry calibration fluorescence intensity which represented the expression strength of target promoter was detected and analyzed.

**FIGURE 5:**
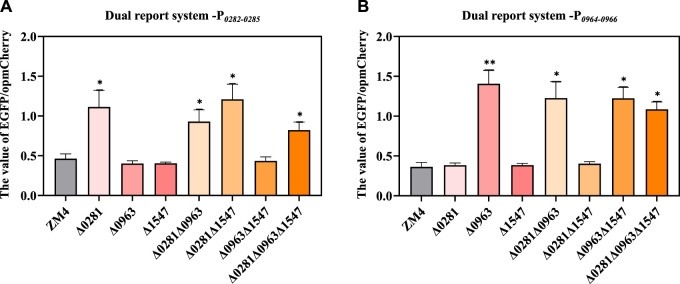
Examination of the regulation of TetR-family regulators on promoter P_*0282-0285*_ **(A)** and P_*0964-0966*_ **(B)** by the dual-reporter system. *t*-test was conducted for the ratio of EGFP/opmCherry with ZM4 as the control, * represents a significant difference (0.01 < *p*-value <0.05), ** represents *p*-value <0.01.

As shown in Figure 5A, P_*0282-0285*_ had an obviously higher EGFP/opmCherry ratio in *ZMO0281* disrupted strains Δ0281, Δ0281Δ0963, Δ0281Δ1547, and Δ0281Δ0963Δ1547 than that of ZM4, while the value of EGFP/opmCherry ratio in strains containing *ZMO0281* such as Δ0963, Δ1547, and Δ0963Δ1547 were similar with ZM4. The knockout of *ZMO0281* released its binding to P_*0282-0285*_ and enabled the higher expression of *EGFP* than ZM4. This was consistent with the conjecture that ZMO0281 was the repressor of *ZMO0282-ZMO0285* by binding to the promoter between *ZMO0281* and *ZMO0282-ZMO0285*. Moreover, ZMO0963 and ZMO1547 rarely participate in the repression of P_*0282-0285*_.

Similarly, P_*0964*-*0966*_ had a significantly higher EGFP/opmCherry ratio in Δ0963, Δ0281Δ0963, Δ0963Δ1547, Δ0281Δ0963Δ1547 compared with ZM4, and the values of EGFP/opmCherry ratios in Δ0281, Δ1547, and Δ281Δ1547 were similar with ZM4 (Figure 5B). This also indicated that ZMO0963 represses P_*0964*-*0966*_. The knockout of the regulator improved the ratio of EGFP/opmCherry. Collectively, the results further confirmed that ZMO0281 and ZMO0963 repress the expression of *ZMO0282-ZMO0285* and *ZMO0964-ZMO0966* by binding to the promoter between *ZMO0281-ZMO0282* and *ZMO0963-0964,* respectively.

### Unravelling of the molecular mechanisms of TetR-family regulators on acetate tolerance

Cell growth measurement suggested that TetR-family regulator ZMO0281 was involved in the acetate tolerance in *Z. mobilis* (Figure 3). To illustrate the underlying mechanism, the detailed gene expression information of different mutant strain comparisons under acetate stress was analyzed, especially the transcriptional profiling in the *ZMO0281* mutants (Supplementary Tables S2-3). The results showed that 47 genes were differentially regulated by *ZMO0281* deletion with Δ0281Δ0963Δ1547 compared with Δ0963Δ1547, including 17 genes upregulated and 30 genes downregulated. Among these genes, *ZMO0282-0285* were the most differentially upregulated genes (more than eight-fold), which were also induced in the mutant strains Δ0281Δ0963, Δ0281Δ1547, and Δ0281Δ0963Δ1547 compared with the wild-type ZM4 as stated earlier.

Besides the RND-family operon gene, four of the upregulated genes by *ZMO0281* deletion were also shared to be abundant in the *ZMO0281* mutants compared with ZM4. *ZMO0678* is annotated as nitroreductase and it was also induced in all three *ZMO0281* mutants. *ZMO1399* encoding fatty acid hydroxylase was upregulated in Δ0281Δ0963 and Δ0281Δ0963Δ1547 by more than 2 folds, and upregulated in Δ0281Δ1547 by 1.99-fold. *ZMO1753* is a ferredoxin-NADP^+^ reductase gene and it was significantly upregulated in Δ0281Δ0963 and Δ0281Δ1547 but only upregulated in Δ0281Δ0963Δ1547 by 1.78-fold. *ZMO0085* is the fourth common gene which encodes methyl-accepting chemotaxis sensory transducer and had a shared upregulation in Δ0281Δ0963. All four genes above were not differentially expressed in the mutant Δ0963Δ1547 compared with ZM4, thus they were also potentially involved in the ZMO0281 directly regulation as *ZMO0282-0285*. Although the majority of characterized TFRs regulate efflux pumps, the TFRs actually regulate various cellular processes such as nitrogen metabolism, lipid metabolism, co-factor metabolism, and cell signaling (Cuthbertson and Nodwell, 2013; Murarka and Srivastava, 2019).

In addition, other 10 upregulated genes by ZM*O0281* deletion were mainly related to cellular energy-costly process, including exodeoxyribonuclease gene *ZMO1401*, ATP-dependent protease gene *ZMO1704*, ATP/cobalamin adenosyltransferase gene *ZMO1495*, phage shock protein PspA gene *ZMO1603*, and two amino acid metabolim related gene *ZMO1603* (encoding protein tyrosine phosphatase) and *ZMO1382* (encoding aspartate aminotransferase family protein). The upregulation of energy-related genes suggested that the decreased cell growth in *Z. mobilis* after *ZMO0281* knockout might be due to the energy imbalance resulted from the highly increased expression of RND-family efflux pump.

Interestingly, four of the downregulated genes in Δ0281Δ0963Δ1547 compared with Δ0963Δ1547 were associated with sucrose hydrolysis, including *ZMO0374* (*sacB*) and *ZMO0375* (*sacC*) encode sucrose hydrolase, *ZMO0934* (*zliE*) encodes the regulation protein of sucrose hydrolase, which activates the expression of *sacB* and *sacC*, and *ZMO0932* (*zliS*) encodes glycoside hydrolase. This indicated that the decreased cell growth after *ZMO0281* knockout may be also related to the repression of sucrose hydrolysis. In addition, *ZMO1792* (*ilvD*) which related to amino acid metabolism was found to be repressed as well (Supplementary Table S3). However, about 20 of the downregulated genes by ZM*O0281* disruption were hypothetical protein genes, which should be elucidated by ZMO0281 regulation in response to acetate stress in the future.

## Conclusion

In this study, three TetR-family regulator genes *ZMO0281*, *ZMO0963,* and *ZMO1547* in *Z. mobilis* were systematically compared and characterized. The BLAST and sequence alignment research identified a higher similarity between *ZMO0281* and *ZMO0963* that different from *ZMO1547*. The genetic loci further discovered that two RND-family efflux pumps are divergently oriented adjacent to the TetR-family regulator gene *ZMO0281* and *ZMO0963*, respectively. Combining RNA-Seq transcriptomic study and the dual-reporter gene system, this study demonstrated that three TetR-family regulators specifically repress their adjacent genes, with *ZMO0281* to *ZMO0282-0285*, *ZMO0963* to *ZMO0964-0966*, *ZMO1547* to *ZMO1543-1545* and *ZMO1548*, and there are few cross-talks between the TetR-family regulator genes. In addition, the genetics and physiological studies were carried out with various mutants of TetR-family regulators constructed, and the study revealed that *ZMO0281* was involved in the acetate tolerance in *Z. mobilis*. RNA-Seq transcriptomic study suggested that ZMO0281 might also be involved in other cellular processes in the presence of acetate, and the highly upregulation of RND-family efflux pumps by *ZMO0281* deletion might lead to an energy imbalance resulting in a decreased cell growth in *Z. mobilis* under acetate stress.

## Data Availability

The datasets presented in this study can be found in online repositories. The names of the repository/repositories and accession number(s) can be found in the article/Supplementary Material.
