# Supplementary material for: Systematic investigation of TetR-family transcriptional regulators and their roles on lignocellulosic inhibitor acetate tolerance in Zymomonas mobilis
Source: Front Bioeng Biotechnol. 2024 Mar 22;12:1385519. doi: 10.3389/fbioe.2024.1385519 (PMC10998469; doi:10.3389/fbioe.2024.1385519)
Supplement: Supplementary file 2 [file DataSheet1.docx]

**Systematic Investigation of TetR-family Transcriptional Regulators and Their Roles to Acetate Tolerance in *Zymomonas mobilis***

Yubei Xiao^1^, Tongjia Qin^1, 2^, Shuche He^1^, Yunhao Chen^1^, Han Li^1^, Qiaoning He^1^, Xia Wang^1*^, Shihui Yang^1^^*^

^1^State Key Laboratory of Biocatalysis and Enzyme Engineering, and School of Life Sciences, Hubei University, Wuhan, 430062, China

^2^Chinese Medicine College, Guangdong Yunfu Vocational College of Chinese Medicine, Guangdong, 527400, China

**E-mail：**

YX: xyb@stu.hubu.edu.cn

TQ: qintongjia@163.com

HS: shuchehe@stu.hubu.edu.cn

YC: chenyunhao@stu.hubu.edu.cn

HL: hanLi@stu.hubu.edu.cn

QH: Qiaoninghe@hubu.edu.cn

XW: [xxwang@hubu.edu.cn](mailto:xxwang@hubu.edu.cn)

SY: Shihui.Yang@hubu.edu.cn

* Corresponding author.

**FIGURE S1** The palindromic sequences predicted to be recognized by TetR-family regulators and the molecular docking energy of three TetR-family regulators to the six potential operators. The negative value represents that the combination is theoretically accessible.

**FIGURE S2** The dual reporter_gene system used to identify the relationship of candidate TetR-family regulators with the corresponding adjacent promotors in *Z. mobilis*. The system includes the reporter gene *opmCherry* driven by the constitutive promoter P*_lavUV5_* as the intrinsic control, and another reporter gene *EGFP* under the control of promoter P*_0282-0285_* or P*_0964-0966_*.

**TABLE S1** Bacterial strains and plasmids used in this study.

| **Strains or plasmids** | **Description** | **Source** |
| --- | --- | --- |
| **Strains** | | |
| ***Z. mobilis*** | | |
| ZM4 | *Z. mobilis* wild type strain | Lab stock |
| ZM4 (pEZ15A) | ZM4 with an empty plasmid pEZ15A | Lab stock |
| Δ0281 | *ZMO0281* knockout mutant of ZM4 | This study |
| Δ0963 | *ZMO0963* knockout mutant of ZM4 | This study |
| Δ1547 | *ZMO1547* knockout mutant of ZM4 | This study |
| Δ0281 (pEZ15A-0281) | Δ0281 with a plasmid pEZ15A-0281 | This study |
| Δ0963 (pEZ15A-0963) | Δ0963 with a plasmid pEZ15A-0963 | This study |
| Δ1547 (pEZ15A-1547) | Δ1547 with a plasmid pEZ15A-1547 | This study |
| ZM4 (pEZ15A-0281) | ZM4 with a plasmid pEZ15A-0281 | This study |
| ZM4 (pEZ15A-0963) | ZM4 with a plasmid pEZ15A-0963 | This study |
| ZM4 (pEZ15A-1547) | ZM4 with a plasmid pEZ15A-1547 | This study |
| Δ0281Δ0963 | *ZMO0281* and *ZMO0963* knockout mutant of ZM4 | This study |
| Δ0281Δ1547 | *ZMO0281* and *ZMO1547* knockout mutant of ZM4 | This study |
| Δ0963Δ1547 | *ZMO0963* and *ZMO1547* knockout mutant of ZM4 | This study |
| Δ0281Δ0963Δ1547 | ZM4 with three TetR genes knockout | This study |
| ZM4 (pEZ-Dual-P*_0282-0285_*) | ZM4 with pEZ-Dual-P*_0282-0285_* | This study |
| ZM4 (pEZ-Dual-P*_0964-0966_*) | ZM4 with pEZ-Dual-P*_0964-0966_* | This study |
| Δ0281 (pEZ-Dual-P*_0282-0285_*) | Δ0281 with pEZ-Dual-P*_0282-0285_* | This study |
| Δ0281 (pEZ-Dual-P*_0964-0966_*) | Δ0281 with pEZ-Dual-P*_0964-0966_* | This study |
| Δ0963 (pEZ-Dual-P*_0282-0285_*) | Δ0963 with pEZ-Dual-P*_0282-0285_* | This study |
| Δ0963 (pEZ-Dual-P*_0964-0966_*) | Δ0963 with pEZ-Dual-P*_0964-0966_* | This study |
| Δ1547 (pEZ-Dual-P*_0282-0285_*) | Δ1547 with pEZ-Dual-P*_0282-0285_* | This study |
| Δ1547 (pEZ-Dual-P*_0964-0966_*) | Δ1547 with pEZ-Dual-P*_0964-0966_* | This study |
| Δ0281Δ0963 (pEZ-Dual-P*_0282-0285_*) | Δ0281Δ0963 with pEZ-Dual-P*_0282-0285_* | This study |
| Δ0281Δ0963 (pEZ-Dual-P*_0964-0966_*) | Δ0281Δ0963 with pEZ-Dual-P*_0964-0966_* | This study |
| Δ0281Δ1547 (pEZ-Dual-P*_0282-0285_*) | Δ0281Δ1547 with pEZ-Dual-P*_0282-0285_* | This study |
| Δ0281Δ1547 (pEZ-Dual-P*_0964-0966_*) | Δ0281Δ1547 with pEZ-Dual-P*_0964-0966_* | This study |
| Δ0963Δ1547 (pEZ-Dual-P*_0282-0285_*) | Δ0963Δ1547 with pEZ-Dual-P*_0282-0285_* | This study |
| Δ0963Δ1547 (pEZ-Dual-P*_0964-0966_*) | Δ0963Δ1547 with pEZ-Dual-P*_0964-0966_* | This study |
| Δ0281Δ0963Δ1547 (pEZ-Dual-P*_0282-0285_*) | Δ0281Δ0963Δ1547 with pEZ-Dual-P*_0282-0285_* | This study |
| Δ0281Δ0963Δ1547 (pEZ-Dual-P*_0964-0966_*) | Δ0281Δ0963Δ1547 with pEZ-Dual-P*_0964-0966_* | This study |
| ***E. coli*** | | |
| DH5α | *E. coli* for plasmid construction | Lab stock |
| Trans110 | *E. coli* for plasmid demethylation | Lab stock |
| **Plasmids** | | |
| pEZ15A | Shuttle vector contains *Z. mobilis* origin and *E. coli* origin p15A; Spe*^R^*; Biobrick-compatible | [1] |
| pEZ15A-0281 | pEZ15A containing *ZMO0281* driven by its native promoter | This study |
| pEZ15A-0963 | pEZ15A containing *ZMO0963* driven by its native promoter | This study |
| pEZ15A-1547 | pEZ15A containing *ZMO1547* driven by its native promoter | This study |
| pL2R | pEZ15A containing a DNA fragment of two tandem copies of CRISPR repeat for artificial CRISPR loci construction | [2] |
| pL2R-0281 | pL2R containing a CRISPR locus with a spacer matching a protospacer in *ZMO0281*, and a donor of recombination arms homologous to the sequences flanking *ZMO0281* | This study |
| pL2R-0963 | pL2R containing a CRISPR locus with a spacer matching a protospacer in *ZMO0963*, and a donor of recombination arms homologous to the sequences flanking *ZMO0963* | This study |
| pL2R-1547 | pL2R containing a CRISPR locus with a spacer matching a protospacer in *ZMO1547*, and a donor of recombination arms homologous to the sequences flanking *ZMO1547* | This study |
| pEZ-Dual | pEZ15A containing reporter gene *opmCherry* driven by P*_lavUV5_* and *EGFP* driven by P*tet* which can be replaced by the target genetic element | [3] |
| pEZ-Dual-P*_0282-0285_* | pEZ-Dual containing P*_0282-0285_* to drive the  reporter gene *EGFP* | This study |
| pEZ-Dual-P*_0964-0966_* | pEZ-Dual containing P*_0964-0966_* to drive the  reporter gene *EGFP* | This study |

**Additional References**

[1] Yang, S., Mohagheghi, A., Franden, M. A., Chou, Y., Chen, X., Dowe, N., et al. (2016). Metabolic engineering of *Zymomonas mobilis* for 2,3-butanediol production from lignocellulosic biomass sugars. *Biotechnol. Biofuels* 9, 189. doi: 10.1186/s13068-016-0606-y

[2] Zheng, Y., Han, J., Wang, B., Hu, X., Li, R., Shen, W., et al. (2019). Characterization and repurposing of the endogenous Type I-F CRISPR-Cas system of *Zymomonas mobilis* for genome engineering. *Nucleic Acids Res.* 47, 11461-11475. doi: 10.1093/nar/gkz940

[3] Yang, Y., Shen, W., Huang, J., Li, R., Xiao, Y., Wei, H., et al. (2019). Prediction and characterization of promoters and ribosomal binding sites of *Zymomonas mobilis* in system biology era. *Biotechnol. Biofuels* 12, 52. doi: 10.1186/s13068-019-1399-6
